# Nonalcoholic Fatty Liver Disease in Chronic Hepatitis B and C Patients from Western Amazon

**DOI:** 10.1155/2012/695950

**Published:** 2012-08-15

**Authors:** A. C. M. Nascimento, D. R. Maia, S. M. Neto, E. M. Lima, M. Twycross, R. F. Baquette, C. M. O. Lobato

**Affiliations:** ^1^Faculty of Medicine, Acre Federal University, 69915-900 Rio Branco, Acre, Brazil; ^2^University of Queensland, Brisbane, St Lucia, QLD 4072, Australia; ^3^Dean of Pathology Lab, Clinical Hospital of Acre, Rio Branco, Acre, Brazil; ^4^Specialized Attendance Service, Clinical Hospital of Acre, Rio Branco, Acre, Brazil

## Abstract

Nonalcoholic fatty liver disease (NAFLD) includes a wide spectrum of histological conditions, extending from simple steatosis to end-stage liver failure. The aim of this study was to examine the prevalence of NAFLD and its associations in chronic hepatitis B and C patients. *Methods*. We included all patients diagnosed with chronic hepatitis B and C who underwent a liver biopsy between January 2010 and October 2011 (*n* = 104). Parameters studied included hepatitis type, anthropometric data, histologic, hepatic, metabolic and lipid assessments, presence of hypertension and viral load. *Results*. Hepatitis B was presented in 28.8% (*n* = 30) of patients, while hepatitis C was presented in 71.2% (*n* = 74). In addition, hepatic steatosis was present in 25% (*n* = 26) of the patients. Steatosis was frequently found in hepatitis C patients (31.1%; 25% *n* = 23), but infrequently in hepatitis B patients (10%; *n* = 3) (*P* = 0.024). It was also found that steatosis was frequently present in hepatitis C patients with intense fibrosis (52.94%) (*P* = 0.025). *Discussion*. Our results suggest that steatosis is a common feature in patients with viral chronic hepatitis, and that it plays a different role in each type of hepatitis.

## 1. Introduction

Nonalcoholic fatty liver disease (NAFLD) includes the whole clinical spectrum of liver damage, from simple steatosis and steatohepatitis to advanced fibrosis and cirrhosis [1]. The diagnosis of nonalcoholic fatty liver disease (NAFLD) requires evidence of fatty changes in the liver in the absence of a history of excessive alcohol consumption [2].

The term nonalcoholic steatohepatitis (NASH) represents a stage within the spectrum of NAFLD. It consists histologically of the presence of steatosis along with necroinflammatory activity, mostly of lobular distribution and regardless of the presence of fibrosis or Mallory's hyaline [1]. The coexistence of NAFLD with other liver diseases, especially with viral hepatitis, such as hepatitis B and C, modifies its natural history and makes diagnosis difficult [3].

NAFLD is often associated with obesity, type 2 diabetes mellitus, and dyslipidemia and is also regarded as a hepatic manifestation of metabolic syndrome [4]. In some recent studies, chronic hepatitis C (CHC) has been shown as a risk factor for NAFLD [5]. However, CHC patients often have features of the metabolic syndrome and a higher prevalence of hepatic steatosis, especially in nongenotype 3 patients.

Genotype 3 is a teatogenic virus and the severity of hepatic steatosis is related to high viral load in the serum as well as high intrahepatic viral load. In this case, steatosis usually resolves with successful antiviral therapy [6, 7]. Viral effects include a decrease of adiponectin levels, and changes to hepatic lipid metabolism that lead to triglyceride accumulation [8, 9]. The incidence of hepatic steatosis in CHC patients ranged from 31% to 72% [10].

In cases of chronic hepatitis B (CHB) infection, the clinical significance of steatosis and its relation to HBV genotypes are unknown [11]. Superimposed NAFLD and NASH in patients with CHB are thought to be related to host factors, especially the metabolic syndrome [12] and may be associated with the increased fibrosis in patients with chronic HBV infection [13]. The frequency of hepatic steatosis in CHB patients is higher than that reported for the general population, but lower than that in CHC patients [14]. It has been shown that the incidence of hepatic steatosis in CHB patients was approximately 32% [12].

This study examines the prevalence of NAFLD in CHC and CHB patients assisted in Acre Hospital of Clinics and also NAFLD association with viral and host factors and with histopathological features.

## 2. Materials and Methods

Acre is a state located in the northern region of Brazil, Western Amazon. The study population currently lives in Western Amazon. This population is highly multicultural, however most residents are descendants from indigenous Western Amazonians.

This study included all patients diagnosed with chronic hepatitis B and C who underwent liver biopsy between January 2010 and October 2011 in Clinical Hospital of Acre (*n* = 130). As a retrospective transversal study, patient records were evaluated. We included all patients with presence of HBV-related chronic liver disease with hepatitis-B-surface-antigen- (HbsAg-) positive for over 6 months. Patients with HCV-related chronic liver disease with positive antibody (anti-HCV), as well as positive qualitative polymerase chain reaction (PCR) were also included. We excluded patients whose records could not be found, patients with alcohol intake >200 g per week, and those who had co-infection with HIV or hepatitis D (*n* = 26). The final number evaluated was 104 patients (*n* = 104).

Clinical variables included were sex, age, hepatitis type, BMI (body mass index), glucose serum level, total cholesterol, HDL (high-density cholesterol), LDL (low-density cholesterol), triglycerides, total protein, albumin, PT (prothrombin time), INR (international normalized ratio) and transaminase levels, the presence of hypertension, and viral load. We also collected information about treatment status (never treated, currently being treated, previously treated, and which treatment scheme). In patients with hepatitis B, we collected serological data (HBsAg, HBeAg, and anti-HBc total). In patients with hepatitis C, we collected genotype. Hepatitis B virus genotype could not be collected because this was not routinely performed clinical patients in the Clinical Hospital of Acre. In addition, all hepatitis B patients were tested for hepatitis D as a usual procedure of the hospital. All the laboratory data was collected from patient records after the date of biopsy.

Pathological data that were collected from biopsy reports included presence of steatosis, presence of fibrosis, steatosis type (micro/macrovacuolar), steatosis area (diffuse or focus), and Metavir score (inflammatory activity and fibrosis). Brazilian Society of Pathology classifications were also collected. This included lobular activity, lobular architecture, parenchymatous activity, and siderosis [15].

We classified fibrosis according to Metavir staging system whereby: F0—absence of fibrosis; F1—portal fibrosis; F2—portal fibrosis with septum; F3—nodular transformation; F4—cirrhosis [16]. Patients were separated into groups according to fibrosis level: soft fibrosis (F0–F2) and advanced fibrosis (F3-F4). Standard pathological methods included were hematoxylin-eosin, Masson's trichrome, and Perl's coloration. For Metavir scoring, at least 6 to 8 portal traits were analyzed.

Statistical analysis was performed using SPSS 13.0 (*Statistic package for social science*) for *windows*. First, we divided the patients in two groups: hepatitis C and hepatitis B. In each group, we performed descriptive statistics (frequency, medium, and standard deviation) and univariate analysis with qui-square test (*χ* ^2^). The Statistical significance level adopted was *P* < 0.05.

This study was completed adhering to ethical aspects from The Helsinki Convention and was approved by Acre Federal University ethics committee.

## 3. Results

Our study analyzed 104 patients. The mean age was 49.00 (±10.70) years. Hepatitis B was present in 30 patients (28.8%) and hepatitis C in 74 patients (71.2%). 56.4% (*n* = 57) of patients were the males and 43.5% (*n* = 44) the females. The mean BMI was 56.4% (*n* = 57) (Table 1).

Hypertension was present in 21.2% (*n* = 22) of the patients. Mean blood sugar level was 95.50 mg/dL (±29.72). Total cholesterol mean was 168.47 mg/dL (±61.41). 

Hepatic steatosis was present in 25% (*n* = 26) of the patients. Steatosis was frequently found in hepatitis C patients (31.1%; *n* = 23). Conversely, steatosis presented only in 10% (*n* = 3) of hepatitis B patients (*P* = 0.024) (Table 2). When hepatitis B was analyzed separately, BMI and triglycerides levels were statistically significant (Table 3). On the other hand, hepatitis C analysis did not show any variable of statistical significance associated with steatosis.

Diffuse steatosis was present in 25% (*n* = 26). In analysis of pathological features, we found that in hepatitis C patients steatosis correlated with increased lobular architecture and parenchymatous activity levels. On the other hand, for patients with hepatitis B, steatosis had no statistically significant association with pathological features.

In 72 hepatitis C patients, we were able to analyse genotype. Genotype 1 was the most frequent with 76.3% (*n* = 55), followed by genotype 3 with 23.6% (*n* = 17). However, steatosis was more common in genotype 3 patients than genotype 1 patients (35.9% versus 30.9%). But this data did not show statistical significance (*P* = 0.736). Diffuse steatosis was found in 72.72%, in which 83.3% of those corresponded to genotype 3. However, this data also did not show statistical significance. Macrovesicular steatosis was found in 80% of patients with steatosis, being more frequently associated with genotype 1.

**Figure 1:**
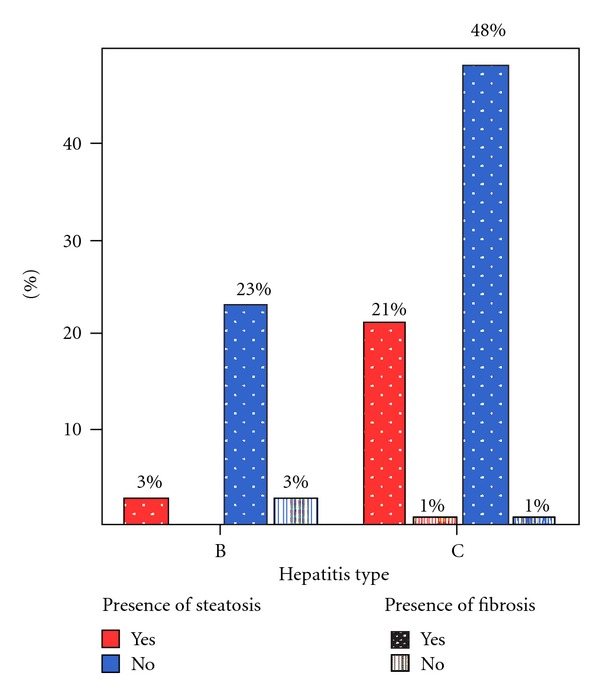
Fibrosis and steatosis rate according to hepatitis type.

With respect to fibrosis levels, steatosis was frequently found in hepatitis C patients with intense fibrosis (52.94%) (*P* = 0.025). In hepatitis B patients, no associations were found, and only 10% presented steatosis (Figure 1). Siderosis was not found in any of the studied patients.

## 4. Discussion

In this study, 10% of hepatitis B patients and 31.1% of hepatitis C patients showed evidence of steatosis. In a previous study of hepatitis B patients, the incidence of steatosis was found to be 32% [12]. The low rate of steatosis in patients from our study is difficult to explain but it may be related to selection of patients for liver biopsy or specific genetic factors in the population of Western Amazon. We also excluded patients with alcohol intake >200 g per week, which has a central role in the development of steatosis. A study performed by Gordon et al. showed a prevalence of steatosis in hepatitis B patients of 76%. However, in this study, alcohol intake patients were included [17].

The rate of steatosis detected in hepatitis C patients was lower compared to other studies that show a variation between 32 and 72% [10]. This data could be explained by the lower prevalence of genotype 3 infection and perhaps by the lower mean of BMI. Therefore, BMI showed statistical significance only in the hepatitis B group, which indicates its relevance in steatosis pathogenesis in hepatitis B patients.

Triglycerides levels were also found to be higher in hepatitis B patients. Although our study was not able to associate other lipid parameters with the presence of steatosis, high levels of triglycerides seem to be a good marker of dyslipidemia. Bondini et al. found correlation between presence of dyslipidemia and hepatic steatosis [12]. However, Rastogi et al. also found association between triglycerides levels and steatosis [11].

Hepatic steatosis is characterized by the accumulation of lipids in hepatocytes and is associated with diverse systemic conditions and various primary liver diseases [18]. In addition, we found that steatosis was frequently found in hepatitis C patients with intense fibrosis (52.94%) (*P* = 0.025), which could indicate a significant relationship between steatosis and fibrosis in hepatitis C patients. Hsieh et al. showed fibrosis as an important risk factor for steatosis [5]. Therefore, pathological parameters such as lobular architecture and parenchymatous activity were related to the presence of steatosis, indicating that pathological hepatic transformation is related to steatosis.

It is known that genotype 3 is causal for hepatic steatosis which usually resolves with successful antiviral therapy [6, 7]. However, our analysis did not show statistical significance between genotype and steatosis, perhaps because of the increased number of individuals with genotype 1.

This study was based on patient records. Consequently, we have to note that some data could have been lost by register errors. However, this is the first study on NAFLD in chronic viral hepatitis patients in Acre state. Further studies in the Western Amazon are required, since it is a region with a high incidence of chronic viral hepatitis.

## Figures and Tables

**Table 1:** Patients characteristics according to hepatitis type.

	Hepatitis B		Hepatitis C		*P*
	%	*N*	%	*N*
Gender					
Feminine	40.9	18	59.1	26	**0.009**
Masculine	17.5	10	82.5	47	
BMI					
>30	18.8	3	81.3	13	0.305
25–29.9	22.2	8	77.8	28	
<25	34.7	17	65.3	32	
Steatosis					
Yes	11.5	3	88.5	23	**0.024**
No	34.6	27	65.4	51	
Fibrosis					
Yes	27.3	27	72.7	72	0.115
No	60.0	3	40.0	2	

**Table 2:** Patients characteristics according to the presence of hepatic steatosis.

	Hepatic steatosis				
	Yes		No		*P*
	%	*N*	%	*N*
Hepatitis					
B	10.0	3	90.0	27	**0** **.024**
C	31.1	23	68.9	51	
Gender					
Feminine	29.5	13	70.5	31	0.442
Masculine	22.8	13	77.2	44	
BMI					
>30	31.3	5	68.8	11	0.255
25–29.9	33.3	12	66.7	24	
<25	18.4	9	81.6	40	
Hypertension					
Yes	31.8	7	68.2	15	0.382
No	22.7	17	77.3	58	
Glycaemia					
>200 mg/dL	0.0	0	100.0	2	0.257
100–199 mg/dL	40.0	8	60.0	12	
<100 mg/dL	24.3	18	75.7	56	
Cholesterol					
>200 mg/dL	25.0	4	75.0	12	0.205
<200 mg/dL	30.8	20	69.2	45	
Triglycerides					
>200 mg/dL	50.0	4	50.0	4	0.133
<200 mg/dL	25.0	18	75.0	54	
Fibrosis					
Yes	25.3	25	74.7	74	0.791
No	20.0	1	80.0	4	

**Table 3:** Hepatitis B individual features according to the presence of hepatic steatosis.

	Hepatic Steatosis				
	Yes		No		*P*
	%	*N*	%	*N*
Gender					
Feminine	11.1	2	88.9	16	0.927
Masculine	10.0	1	90.0	9	
BMI					
>30	0.0	0	100.0	3	**0**.**015**
25–29.9	37.5	3	62.5	5	
<25	0.0	0	100.0	17	
Hypertension					
Yes	16.7	1	83.3	5	0.623
No	9.5	2	90.5	19	
Glycaemia					
>200 mg/dL	0.0	0	0.0	0	0.713
100–199 mg/dL	0.0	0	100.0	1	
<100 mg/dL	12.0	3	88.0	22	
Cholesterol					
>200 mg/dL	0.0	0	100.0	8	0.224
<200 mg/dL	16.7	2	83.3	10	
Triglycerides					
>200 mg/dL	50.0	1	50.0	1	**0**.**047**
<200 mg/dL	5.6	1	94.4	17	
